# Supplementary material for: Inflammatory factors and risk of lung adenocarcinoma: a Mendelian randomization study mediated by blood metabolites
Source: Front Endocrinol (Lausanne). 2024 Aug 27;15:1446863. doi: 10.3389/fendo.2024.1446863 (PMC11384989; doi:10.3389/fendo.2024.1446863)
Supplement: Supplementary file 1 [file DataSheet1.zip › supplementary files/S2/GCST90200247/sensitivity-analysis.pdf]

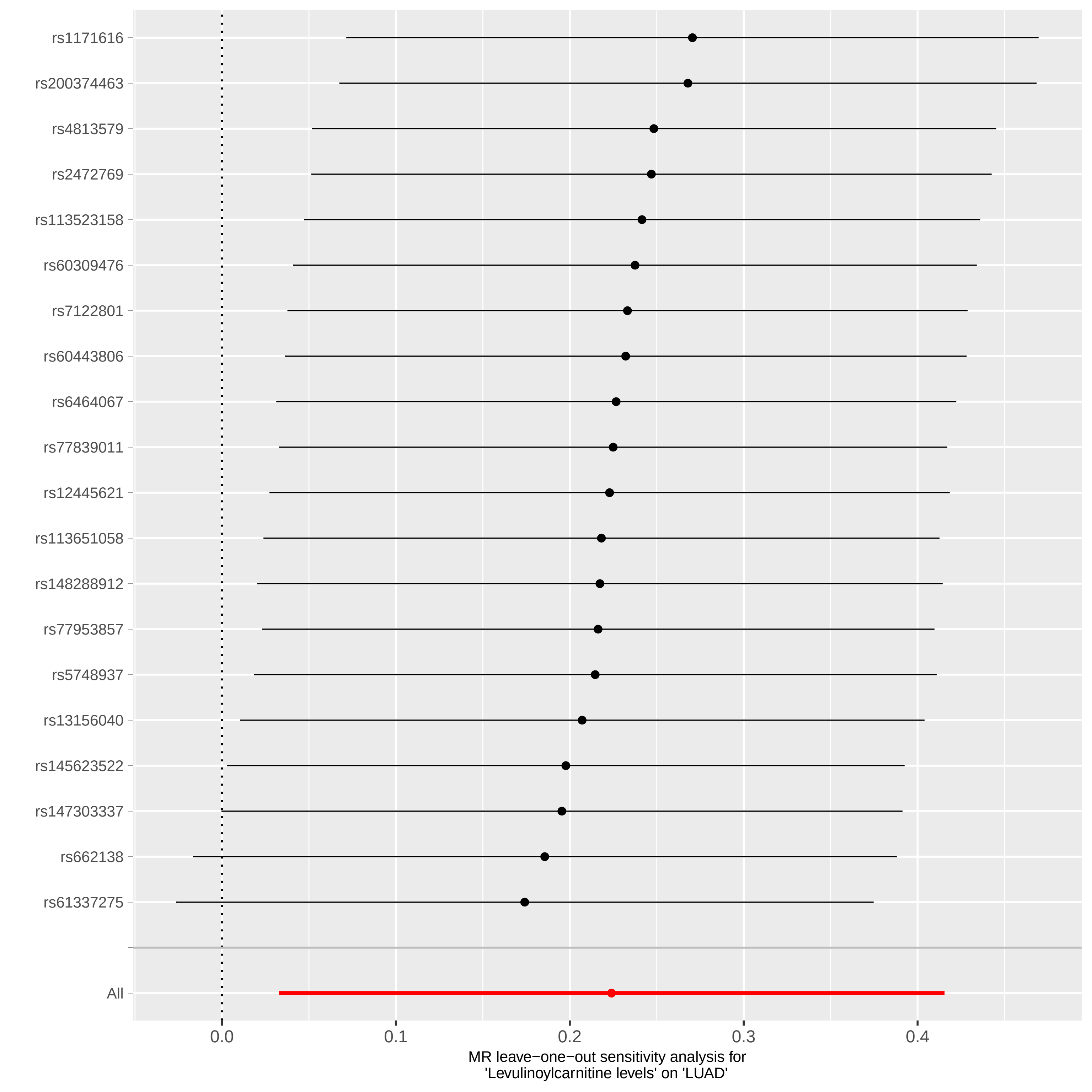

rs1171616
rs200374463
rs4813579
rs2472769
rs113523158
rs60309476
rs7122801
rs60443806
rs6464067
rs77839011
rs12445621
rs113651058
rs148288912
rs77953857
rs5748937
rs13156040
rs145623522
rs147303337
rs662138
rs61337275
All
0.0
0.1
0.2
0.3
0.4
MR leave−one−out sensitivity analysis for
'Levulinoylcarnitine levels' on 'LUAD'
